# Diagnosis and Analysis of Vasa Previa Types With Flow HD Glass Body

**DOI:** 10.1002/jum.16595

**Published:** 2024-10-03

**Authors:** Lili Gong, Lipeng Zheng, Junhui Gao, Hongbo Chang, Ying Liu, Yingluan Wang

**Affiliations:** ^1^ Department of Ultrasound Zibo Municipal Hospital Zibo China

**Keywords:** color Doppler, glass body, prenatal, ultrasound examination, umbilical vein, vasa previa

## Abstract

**Objectives:**

To explore the value of applying flow high definition (HD) glass body in prenatal diagnosis of vasa previa and to preliminarily discuss the types of vasa previa.

**Methods:**

Two‐dimensional ultrasound, flow HD, and flow HD glass body were used to image the umbilical cord insertion site and placenta, observe the cervical internal os and surrounding areas, and retrospectively analyze cases of vasa previa.

**Results:**

There were 15 cases of vasa previa, including 14 cases of singleton pregnancies and 1 case of twin pregnancy, with a total of 22 vasa previa, including 10 veins and 12 arteries. There was 1 case with 3 vessels, 5 cases with 2 vessels, and 9 cases with a single vessel. Among them, in 3 cases of vasa previa detected at 12, 14, and 24 weeks, respectively, the vasa previa were relocated to a normal position at 24, 29, and 35 weeks of gestation when re‐examined. Routine 2‐dimensional ultrasound examination in this group showed tubular or circular hypoechoic areas near the cervical internal os, but vasa previa could not be confirmed. Flow HD could display color blood flow at and near the cervical internal os in 15 cases, but it was difficult to continuously show the course and source of the blood vessels under the chorion. Flow HD glass body from multiple angles could display the relationship between 15 cases of 22 vasa previa and the placenta and cervix. Combined with color Doppler blood flow spectra, flow HD glass body could determine the types of vasa previa.

**Conclusions:**

Flow HD glass body imaging can clearly display vasa previa, showing their origin and the spatial relationship with the cervix and placenta in a 3‐dimensional manner, displaying the course and attachment points of umbilical vessels under the chorion. It can observe the area of interest at any angle, and combined with color Doppler blood flow spectra, it can judge the vasa previa of the umbilical vein, providing a more definite imaging basis for clinical management.

Flow high definition (HD) glass body is an imaging mode that combines grayscale ultrasound in glass body mode with high‐resolution color Doppler blood flow (flow HD), commonly used for blood flow imaging in the heart and vessels.^1^ Due to the umbilical cord insertion point and placental vessels being unaffected by fetal movement, volume data of the umbilical cord and placental vessels are easily obtained. Flow HD glass body mode is advantageous for observing abnormalities in the insertion and course of umbilical cord vessels. In cases of vasa previa, the vessels may be either umbilical arteries or umbilical veins. When the vasa previa originate from the umbilical arteries, confirmation can be made by measuring the blood flow spectrum, which should match the fetal heart rate frequency. However, identification of the source of the vessels becomes challenging when the vasa previa are from the umbilical veins, as they are difficult to distinguish from maternal vessels. This study utilizes flow HD glass body mode to display the origin of vasa previa prenatally and their relationship with the placenta and cervix. Combined with color Doppler blood flow spectra, it aims to determine the type of vasa previa, providing valuable imaging evidence for clinical diagnosis and treatment.

## Materials and Methods

### Study Population

A retrospective analysis was conducted on 15 cases of vasa previa diagnosed via ultrasound examination at Zibo Municipal Hospital from January 2020 to December 2023. This was a retrospective single‐center study approved by the hospital's ethics committee (approval number: 20190304). All cases of prenatal ultrasound‐detected vasa previa were included, with maternal ages ranging from 26 to 43 years (mean age: 31.67 ± 4.78 years) and gestational ages ranging from 12 to 24 weeks (mean gestational age: 20.21 ± 4.06 weeks).

### Instruments and Methods

The GE Voluson E10 ultrasound imaging system was used with abdominal 3D volume probes RM6C and eM6c G2, operating at frequencies of 4–8 MHz. Ultrasound examinations were performed following standardized procedures for prenatal ultrasound examination. Routine 2‐dimensional ultrasound examinations were conducted for the placenta and umbilical cord, observing their positions, umbilical cord insertion points, and the cervix. Flow HD was used to observe blood flow at the umbilical cord insertion point and the cervix, and blood flow spectra were measured at the cervix. Before collecting volume data, a prescan was conducted to optimize color flow imaging, maintaining high frame rates and color persistence to improve blood flow visualization in the region of interest, and to collect as much color information as possible. Three‐dimensional volume data were collected using color or power Doppler mode with 3‐dimensional ultrasound glass body mode, encompassing the umbilical cord insertion point, the placenta near the insertion point, and the cervix to fully display the relationship between the sail‐like vessels and the cervix. It took approximately 1–2 seconds to collect one image, and typically, 3 images were collected. The obtained 3‐dimensional volume data were stored in the ultrasound diagnostic instrument or offline workstation, adjusting the appropriate image blending display ratio to highlight grayscale volume imaging or color flow volume imaging for better display of 3‐dimensional ultrasound glass body mode images. Images were rotated along the *x*‐, *y*‐, and *z*‐axes to observe suspected vessel 3‐dimensional images and their relationships with the placenta and cervix at different sections and angles.

Two‐dimensional ultrasound, flow HD, and flow HD glass body were used to image the umbilical cord insertion and the placenta, observing the cervical internal os and its surroundings. vasa previa was diagnosed when sail‐like vessels traversed the fetal membranes within 2 cm of the cervical os.^2^

All cases of vasa previa in this group were confirmed by postnatal follow‐up results.

## Results

There were 15 cases of vasa previa, including 14 singleton pregnancies and 1 twin pregnancy. Among these, there was 1 case with 3 vessels, 5 cases with 2 vessels, and 9 cases with a single vessel. There were a total of 22 vasa previa, including 10 veins and 12 arteries. Among the 15 cases of vasa previa, 13 cases were type I and 2 cases were type II. Among the 3 cases initially diagnosed with previa at 12, 14, and 24 weeks, respectively, the vasa previa were relocated to a position greater than 2.0 cm from the cervical os when re‐examined at 24, 29, and 35 weeks. Routine 2‐dimensional ultrasound examination in this group showed tubular or circular hypoechoic areas near the cervical os, but vasa previa could not be confirmed. Flow HD could display color blood flow at and near the cervical os in 15 cases, but it was difficult to continuously show the course and source of the blood vessels beneath the fetal membrane. Flow HD glass body from multiple angles could display the relationship between 15 cases of 22 vasa previa and the placenta and cervix.

**Figure 1:**
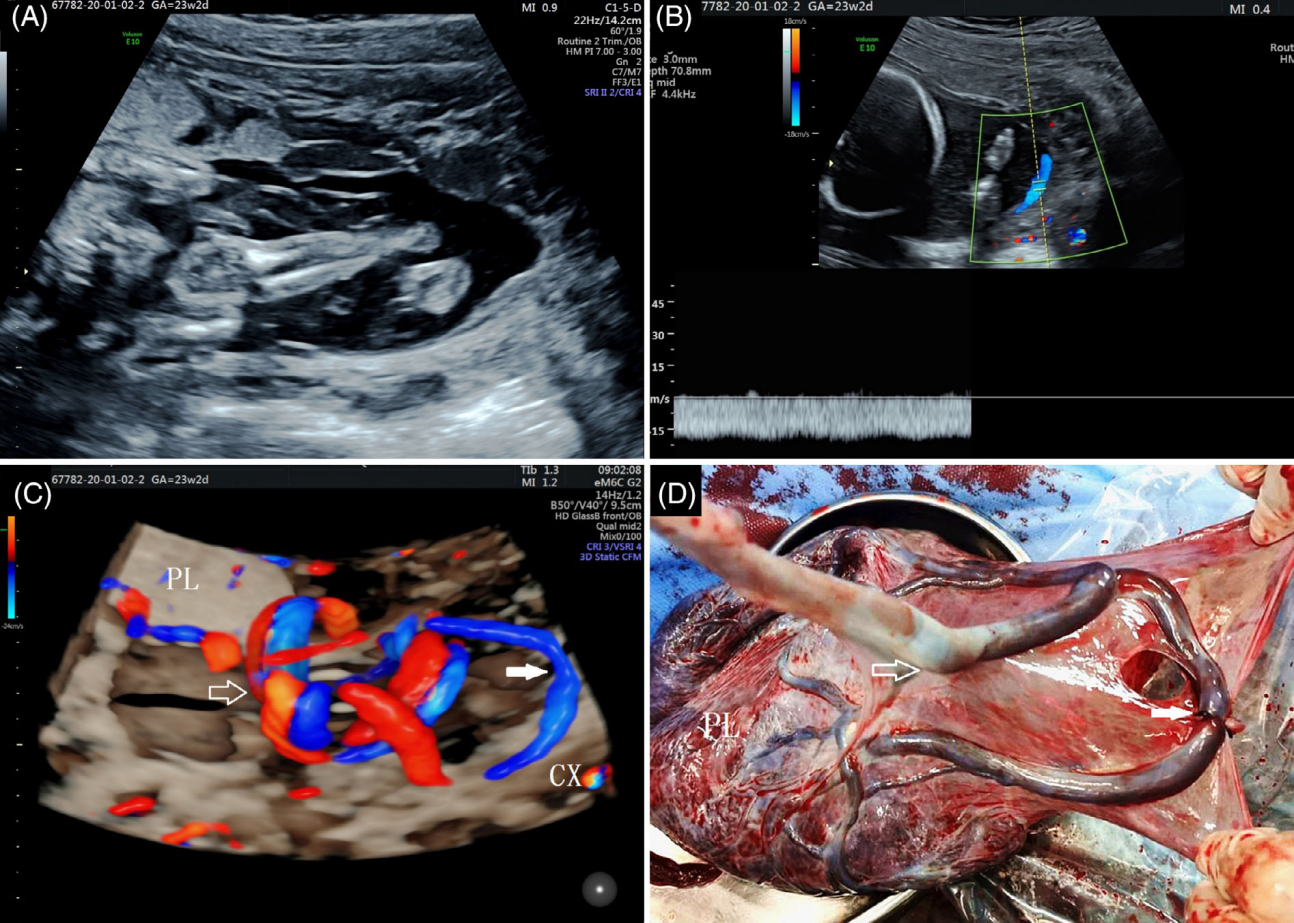
Two‐dimensional ultrasound showing tubular hypoechoic structure near the cervical os suggesting vessel previa (**A**). Color Doppler imaging demonstrating venous blood flow spectrum near the cervical os (**B**). Flow HD glass body of the umbilical cord showing sail‐like insertion and vessel previa through the cervix (**C**). PL, placenta; CX, cervix; hollow arrow, umbilical cord insertion point; solid arrow, sail‐like vessel. Postpartum image of placenta, umbilical cord, and umbilical vessels showing sail‐like insertion point (**D**), PL, placenta; hollow arrow, umbilical cord insertion point; solid arrow, sail‐like vessel.

**Figure 2:**
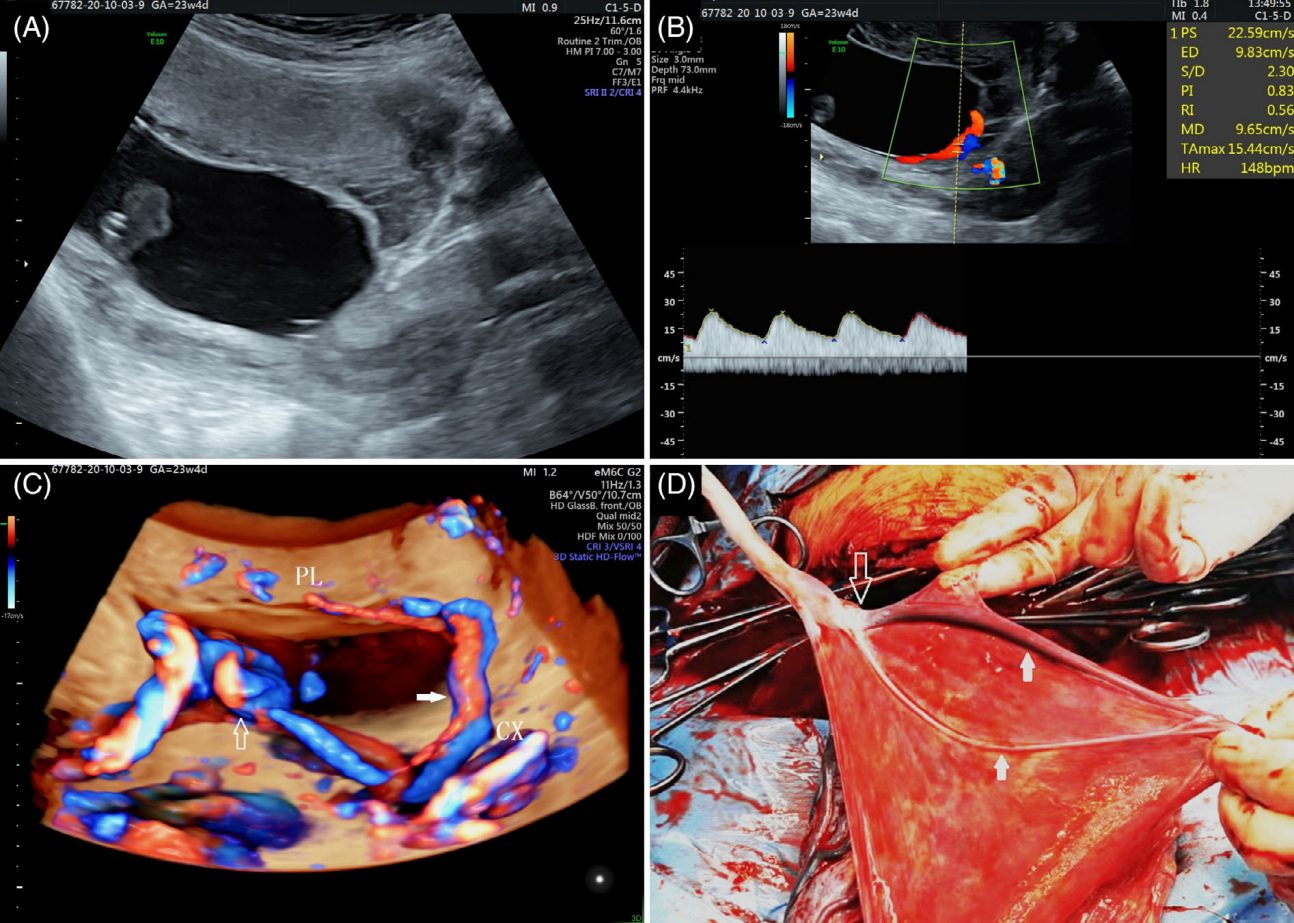
Two‐dimensional ultrasound examination showing short tubular hypoechoic structure near the cervical os suggestive of vessel previa (**A**). Color Doppler imaging demonstrating arterial and venous blood flow spectrum at the cervical os (**B**). Flow HD glass body of the umbilical cord showing sail‐like insertion and vessel previa crossing the cervical os and entering the placenta (**C**). PL, placenta; CX, cervix; hollow arrow, umbilical cord insertion point; solid arrow, sail‐like vessel. Postpartum image of placenta, umbilical cord, and umbilical vessels showing sail‐like insertion point (**D**). PL, placenta; hollow arrow, umbilical cord insertion point; solid arrow, sail‐like vessel.

Performance of flow HD glass body for vasa previa: It can visually display the umbilical cord and its insertion point, with the free umbilical cord appearing helical and located within the amniotic fluid, with variable positions and shapes. Umbilical vessels under the chorion run dispersedly and relatively straight under the fetal membranes, accompanied or unaccompanied by arteries and veins, with fixed positions and no helix. When there is vasa previa, one or several color vessels cross or approach the cervical os, then continue to traverse under the fetal membranes and enter the placenta. The cervix and placenta show a relatively thick dense echo. The umbilical cord, umbilical vessels under the chorion, and the cervix and placenta can be displayed simultaneously (Figures 1 and 2).

## Discussion

Characteristics of vasa previa include unprotected umbilical cord vessels passing through or near the internal cervical os. They are prone to rupture, leading to excessive fetal hemorrhage and potentially resulting in fetal or neonatal death. Compression of umbilical vessels under the chorion can cause fetal hypoperfusion, acidosis, as well as asphyxia, stillbirth, or neonatal death.^3^ Catanzarite divided vasa previa into 2 types based on the morphology of the placenta,^4^ Suekane classified vasa previa cases that were neither like type I nor type II as type III.^5^ Antenatal detection of vasa previa results in a fetal survival rate exceeding 95%. In cases where it is missed antenatally, the perinatal mortality rate exceeds 60%.^6^ Studies suggest that when the vasa previa originate from the umbilical artery, confirmation can be achieved by assessing vessel pulsation with pulse Doppler ultrasound, consistent with fetal heart rate.^7^ At present, other ultrasound display methods are usually difficult to visually show the source and course of vasa previa, particularly when the vessels are umbilical veins, making it difficult to determine whether the vessels originate from the mother or the fetus. Some studies distinguish whether the venous blood vessel is from the mother or the umbilical cord by whether the blood flow pulsates when the pregnant woman takes a deep breath. The blood flow without pulsation is considered to be from the fetal umbilical blood vessel, and the blood flow that pulsates with the pregnant woman taking a deep breath is considered to be from the maternal venous blood vessel.^8^ The course of blood vessels generally has three‐dimensional characteristics. Flow HD is mainly used to simply display blood vessels, while flow HD glass body mode can simultaneously display blood vessels and their surrounding tissue structures. The combination of the two can better display the spatial structure of blood vessels and the spatial relationship between surrounding tissues. Therefore, flow HD glass body can clearly display the relationship between the umbilical blood vessel branches and the internal cervical os, the umbilical cord insertion point, and the placenta, making it easy to establish the diagnosis of vasa previa, especially when there is a high value in the case of umbilical vein vasa previa, and it is more intuitive and easier for the display and classification of various types of vasa previa.

Differentiation diagnosis includes maternal cervical varicose veins located in the lower segment of the uterus or cervix, branching gradually from bottom to top without connection to the umbilical cord or placenta. Presentation of the umbilical cord prolapse: umbilical cord vessels are located below the fetal head, and flow HD glass body shows umbilical cord vessels near or within the cervical canal, with vessels closely accompanying and spiraling. In contrast, vasa previa manifest as vessels located beneath the fetal membranes, with a fixed position and straighter course, passing through or near the internal cervical os before entering the placenta.

Prenatal diagnosis of sail‐shaped cord insertion and vasa previa is effective during mid‐pregnancy (20–24 weeks), with approximately 20% of cases diagnosed during mid‐pregnancy showing migration to normal position before delivery. The earlier the gestational week when vasa previa is detected for the first time, the greater the possibility of migration of vasa previa.^9^ Three cases of vasa previa diagnosed between weeks 13–24 in our study were confirmed to have migrated to a position more than 2 cm away from the internal cervical os by week 35, as verified postpartum.

Flow HD glass body is affected by factors such as oligohydramnios and late pregnancy fetal head descent. Oligohydramnios causes cord aggregation, complicating the relationship between the vessels, requiring careful observation from multiple angles to distinguish between free‐floating umbilical cord and cord insertion point, as well as umbilical vessels under the chorion. Late pregnancy fetal head descent affects the visualization of vasa previa in abdominal ultrasound, but can be addressed by vaginal or perineal ultrasound examination.

## Conclusion

Prenatal diagnosis of vasa previa is crucial for fetal prognosis. Flow HD glass body can display vasa previa, especially identifying the origin of vessels such as umbilical veins in relation to the cervix and placenta's 3‐dimensional morphology and spatial relationships. It can show the course and attachment points of umbilical vessels under the chorion and allows observation from any angle. Combined with color Doppler flow spectrum, it can differentiate types of vasa previa, providing clear imaging evidence for clinical management and effectively preventing adverse outcomes.

## Data Availability

The data that support the findings of this study are available on request from the corresponding author. The data are not publicly available due to privacy or ethical restrictions.
